# Role of endoscopic ultrasound as a predictor of histological healing in ulcerative colitis

**DOI:** 10.1080/07853890.2025.2499961

**Published:** 2025-04-30

**Authors:** Jin Tian, Wei Wang, Yongshuai Liu, Xin Zhang, Hanqing Zhao, Hongmei Qu

**Affiliations:** a School of Clinical Medicine, Shandong Second Medical University, Weifang, Shandong, China; b Department of Gastroenterology, the First Affiliated Hospital of Shandong Second Medical University, Weifang People’s Hospital, Weifang, Shandong, China

**Keywords:** Endoscopic ultrasound, ulcerative colitis, histological healing, Mayo endoscopic score, predictive value

## Abstract

**Background:**

Ulcerative colitis (UC) is a chronic inflammatory bowel disease with rising global prevalence.Histological healing (HH) is a key treatment target associated with better long-term outcomes. Although endoscopic ultrasound (EUS) is known to be related to both clinical and endoscopic activity in UC, its role in defining HH remains unclear. Therefore, this study was aimed at investigating the association between EUS and histological activity (HA), as well as the predictive potential of EUS for HH.

**Method:**

In this cross-sectional analysis, 68 UC adults underwent EUS and colonoscopy with biopsies. We used the Mayo Endoscopic Score (MES) for endoscopic activity, the Nancy Index (NI) for biopsy grading, and the Endoscopic Ultrasound-Ulcerative Colitis (EUS-UC) score for EUS analysis, defining endoscopic remission as MES ≤ 1 and HH as NI ≤ 1.A receiver operating characteristic (ROC) curve was employed to evaluate the ability of the indices to predict HH.

**Results:**

Totally 23 patients (33.80%) achieved HH, while 45 (66.20%) showed HA. The EUS-UC scores were significantly lower in the HH group (*p* < 0.001) and correlated strongly with NI (ρ = 0.73). EUS-UC score was an independent risk factor for HH (adjusted OR = 1.918, 95% CI: 1.195–3.080, *p* = 0.007). The EUS-UC score demonstrated a strong predictive value for HH, with an AUC of 0.840, a sensitivity of 75.56%, and a specificity of 78.26%.

**Conclusion:**

The EUS-UC score has a significant correlation with histological outcomes and shows strong potential as a reliable, invasive predictor of HH in UC, with implications for improved disease monitoring.

## 1. Introduction

Ulcerative colitis (UC) is a diffuse nonspecific inflammatory disorder affecting the rectum and colon to different degrees. In 2023, the global incidence of UC was estimated to be 5 million cases, with a continuing increase in incidence globally [1]. The clinical course of UC is featured by relapses and remissions, which can potentially cause organ impairment and negatively impact quality of life. Although achieving endoscopic remission is a long-term therapeutic target for the treatment of UC [2], studies have indicated that inflammation may still be present microscopically even if endoscopy results remain normal [3,4]. Histological healing (HH) provides better disease control by completely removing microscopic inflammation, including neutrophil infiltration and abnormalities in crypt structure [5,6]. HH not only reduces clinical recurrence rates by over 50% within one year, but also lowers the risk of hormone dependency and hospitalization by approximately 60% during long-term follow-up [7]. In addition, it mitigates cancer risk, as individuals with ongoing inflammation are 2.5 times more likely to develop cancer relative to those with endoscopic mucosal healing (EH) [8,9]. A review also suggested that HH may provide a more precise evaluation of mucosal recovery, a stronger correlation with patient prognosis, as well as a greater ability to predict treatment response and prognostic outcomes [10]. This is why HH, which aims for a deeper stage of healing, is increasingly important [2].

HH of UC can be evaluated through a variety of methods, including biomarkers, Magnetic resonance imaging (MRI), bowel ultrasound, colonoscopy, and histological biopsy. However, each method has its drawbacks. For example, C-reactive protein (CRP), erythrocyte sedimentation rate (ESR), and albumin lack sensitivity, and systemic inflammation or malnutrition can influence these biomarkers [11]. Fecal calprotectin (FC) is costly and cannot localize inflammation effectively [11,12]. Colonoscopy allows only surface observation of mucosal inflammation, and biopsies carry risks of serious complications including bleeding or perforation. MRI is expensive, requires high patient compliance, and struggles to identify mucosal erosions owing to its imaging limitations [13]. Additionally, intestinal ultrasound can be impacted by intestinal gases and peristalsis [6]. Therefore, there is a pressing need for improved tests to evaluate HH in UC. However, owing to endoscopic ultrasound (EUS)’s high-resolution, real-time imaging capabilities at the gastrointestinal wall level, and better localization of inflammation, it is increasingly used for intestinal examinations. As an emerging inspection tool, EUS is widely used to assess gastrointestinal cancer, and benign gastrointestinal, pancreatic and biliary diseases [14]. Recently, several reports indicate that EUS is capable of detecting inflammation in UC patients [15,16]. Particularly, increased total wall thickness (TWT) is related to clinical, endoscopic and histological severity in patients with UC [17]. Yan et al. developed the endoscopic ultrasound-ulcerative colitis (EUS-UC) score, which includes TWT, depth of inflammation and hyperemia [16], and the scoring system was deemed a valuable tool for accurately clarifying the disease condition of patients with UC, assessing the severity of disease and the therapeutic response [18]. However, there appears to be scarce research regarding the correlation between EUS-UC score and histological outcomes in UC patients.

Based on the provided research background, this study was aimed at evaluating the associations between the EUS-UC score and several key biomarkers, endoscopic disease activity and histology. This study introduced a new tool for evaluating HH in UC and established a crucial basis for optimizing patient management strategies. And the application of this scoring system is expected to enhance personalized therapeutic decision-making for UC patients and improve their clinical outcomes.

## 2. Materials and methods

### 2.1. Patients

This study was designed as a cross-sectional analysis to evaluate the diagnostic performance of the EUS-UC score in predicting HH in patients undergoing UC. Through consecutive sampling, we enrolled totally 227 adult UC patients (18 to 80 years) from the Department of Gastroenterology, the First Affiliated Hospital of Shandong Second Medical University (a Comprehensive Tertiary Hospital), between February 2022 and February 2024. Patients were excluded based on the following criteria: (1) contraindications to colonoscopy, including severe cardiopulmonary insufficiency, shock, and fulminant colitis, due to the increased risk of complications. Pregnant patients were also excluded, as colonoscopy and EUS may pose risks to the fetus; (2) inadequate bowel preparation, as poor visualization of the colonic mucosa can result in inaccurate assessments; (3) gastrointestinal malignancy, since the presence of malignancy may alter colonic wall structure and influence EUS-UC scoring; (4) history of colectomy, which can alter anatomy and impact colonoscopy results; (5) incomplete clinical data, ensuring the accuracy and reliability of the analysis; (6) absence of EUS examination, as the EUS-UC score cannot be calculated without EUS data.

**Figure 1.**
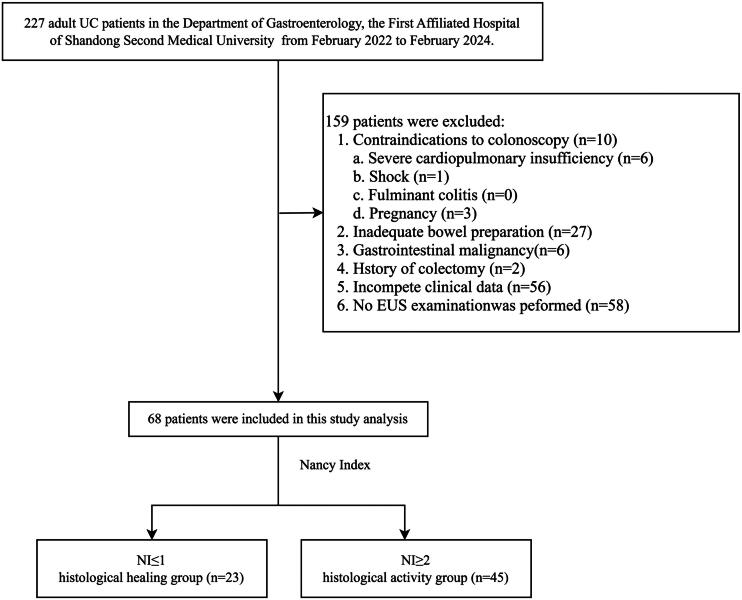
Flowchart. Patient selection process.

Among 227 eligible patients, 68 patients with UC were included and underwent coloscopy and EUS. Subsequently, based on the Nancy index (NI), the included participants will be categorized into two groups including the HH group (NI ≤ 1) and the histological activity (HA) group (NI ≥ 2). Figure 1 presents the steps for including and excluding patients from the study. The current study was performed in accordance with the Declaration of Helsinki, and the Institutional Review Board of the First Affiliated Hospital of Shandong Second Medical University reviewed and approved the study protocol (institutional approval number: KYLL20240327-1). Since this research was a retrospective analysis, the Institutional Review Board of the First Affiliated Hospital of Shandong Second Medical University waived informed consent.

### 2.2. Clinical and laboratory data

The patient’s baseline features, including age, gender, disease duration, therapy, and blood results like erythrocyte sedimentation rate (ESR), albumin, C-reactive protein (CRP), hemoglobin, and the neutrophil-to-lymphocyte ratio (NLR), were collected before the first endoscopic exam. In addition, we also evaluated the Truelove and Witt score based on stool frequency, hematochezia, pulse, ESR, temperature, and hemoglobin, classifying it as remission (0), mild (1), moderate (2), and severe (3).

### 2.3. Endoscopic examination procedure

**Figure 2.**
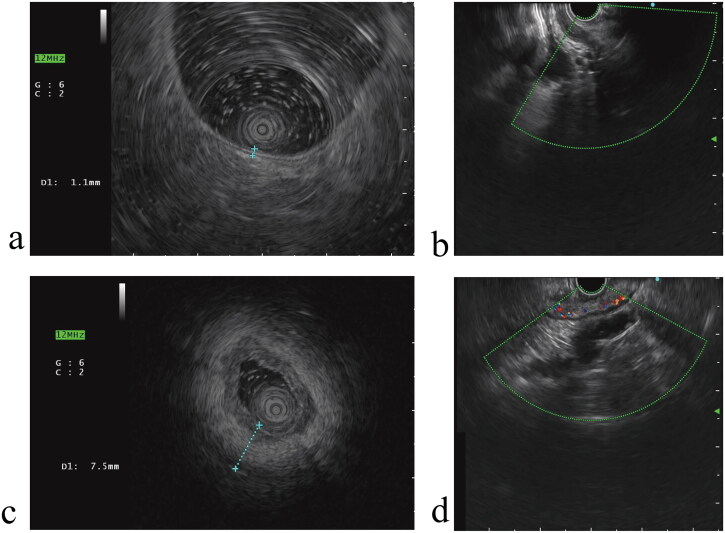
The EUS pictures of patient with UC: total EUS-UC score 0 (lowest score): (a) total wall thickness score 0, depth of inflammation score 0, (b) hyperemia score 0; total EUS-UC score 8 (highest score): (c) total wall thickness score 3, depth of inflammation score 3, (d) hyperemia score 2.

All the enrolled UC patients were fed a liquid diet the day before the endoscopic examination, fasted on the day of the inspection, and were given polyethylene glycol for bowel preparation. Only after adequate bowel preparation, did UC patients underdo colonoscopy and EUS. Endoscopists employed a colonoscope (CF-HQ290I, Olympus, Tokyo, Japan) to enter the ileocecal region and determined the extent of the lesions and endoscopic activity while withdrawing the colonoscope. Concurrently, 3-5 pathological biopsies were performed on the most severe lesions or on the sigmoid colon or rectum in the absence of lesions to determine the HA. Then, EUS examinations were conducted using sector scanning endoscopic ultrasound (EU-ME2, Olympus, Tokyo, Japan) to measure TWT and capture images of hyperemic regions through the Doppler mode. The examination employed the direct contact method, where the water-filled balloon at the tip of the echoendoscope was completely deflated to allow direct contact with the intestinal mucosa. This examination focused on the area next to the sites where pathological biopsies were taken. A 12 MHz frequency was applied to measure wall thickness and analyze layers, enabling a scanning depth of 4 cm. Doppler mode operated at a frequency of 7.5 MHz and a scanning depth of 6 cm was used to assess blood flow characteristics. For further analysis and review, all images were stored in DICOM format (Figure 2).

### 2.4. Endoscopic assessment

All the patients underwent colonoscopy performed by an endoscopist with at least 5 years of clinical experience; the Mayo endoscopic score (MES) was used to assess the endoscopic activity, and the extent of the lesion was evaluated by the Montreal criteria. The MES [19] was categorized into four levels of severity, ranging from 0 to 3 (grade 0: normalization or remission; grade 1: mild activity; grade 2: moderate activity; and grade 3: severe activity). MES ≤ 1 represented endoscopic remission and MES ≥ 2 indicated endoscopic activity. The severity of lesions was categorized into proctitis (E1), left-sided colitis (E2), and extensive colitis (E3) utilizing Monreal.

### 2.5. EUS assessment

All the EUS images were randomly assigned to an experienced endoscopic gastrointestinal ultrasound specialist for evaluation, who was blinded to clinical activity and colonoscopy results. The EUS-UC score [20] was used to assess intestinal TWT, depth of inflammation and hyperemia, as displayed in Table 1.

**Table 1.** Endoscopic ultrasound ulcerative colitis (EUS-UC) score [20].

Item and score	Component
Total wall thickening	
0	Normal: ≤3mm
1	Mild: 3.1-4.0mm
2	Moderate: 4.1-6.0mm
3	Severe: ≥6mm
Depth of inflammation	
0	Superficial: absence of disruption in the 5-layer echo pattern
1	Subepithelial: disruption not beyond 3 layers.
2	Deep: disruption not beyond 4 layers
3	Transmural: disruption to the 5^th^ layer or beyond
Hyperemia	
0	Normal: no signal
1	Mild: discontinuous signal
2	Moderate: consecutive signal
3	Severe: immediate continuous signal

### 2.6. Histological assessment

All the biopsy findings were evaluated by an experienced gastroenteropathy pathologist who was unaware of the clinical activity, colonoscopy findings and EUS-UC score. The NI was employed to grade the obtained biopsies: Grade 0 had few chronic inflammatory cells, Grade 1 showed moderate to severe chronic inflammation, Grade 2 indicated mild acute inflammation, Grade 3 reflected moderate to severe acute inflammation, and Grade 4 involved ulceration or erosion [21]. HH was defined as NI ≤ 1, and HA defined as NI ≥ 2.

### 2.7. Post-hoc power analysis

Prior to the study, a formal power analysis was not conducted. To assess the statistical power of our study considering the observed effect sizes, a post-hoc power analysis was conducted. This analysis aimed to determine whether the sample size of 68 patients was sufficient to predict the ability to access HH.

### 2.8. Sensitivity analysis and multiple imputation

To evaluate the potential implications of excluding 56 patients due to incomplete data, a sensitivity analysis was performed to compare the baseline characteristics between the included (*n* = 68) and excluded (*n* = 56) cohorts. In addition, key demographic and clinical variables were systematically compared.

To further mitigate the potential impact of missing data, multiple imputation was conducted using SPSS 25 software (IBM, New York, USA). The multiple imputation procedure employed the Fully Conditional Specification (FCS) method, iteratively imputing missing values based on the observed data for each variable. The imputation model incorporated the following variables: Age, disease duration, CRP, ESR, albumin, NLR, Truelove and Witt score. For the imputed data, univariate and multivariate logistic regression analysis was used to determine influencing factors and conduct ROC curve analysis to evaluate the diagnostic performance of the EUS-UC score in predicting HH.

### 2.9. Statistical analysis

The statistical analyses were performed with IBM SPSS Statistics 25 software (IBM, New York, USA), and MedCalc 22 software (MedCalc Software Ltd, Ostend, Belgium) was used for plotting the receiver operating characteristic (ROC) curves. GraphPad Prism 9.0 software (GraphPad Software, Boston, USA) was applied to accomplish the visualization process. A post-hoc power analysis was conducted using PASS 2023 (NCSS LLC, Kaysville, Utah, USA). Continuous variables with normal distribution were described by mean and standard deviation (SD), while others used the median and interquartile ranges (IQR). Ordered categorical variables were represented by frequency counts and percentages. The independent samples t-test compared normally distributed continuous variables, and the Mann-Whitney U test was adopted for comparing ordered categorical variables and non-normally distributed data. In addition, correlation analysis was carried out using Spearman’s rank correlation coefficient. To identify independent predictors of HH, univariate logistic regression was firstly performed for variable. Variables with *p* < 0.10 in univariate analysis entered multivariate analysis using backward stepwise regression with likelihood ratio (LR) tests. The ROC curve assessed indices’ ability to predict HH, with the cut-off value being determined by the highest Youden index. Then, to evaluate the predictive accuracy, the area under the ROC curve (AUC), sensitivity and specificity were employed. Statistical significance was defined as *p* < 0.05.

## 3. Results

### 3.1. Clinical characteristics

A total of 68 UC patients, 31 men (45.6%) and 37 women (54.4%) were involved in this study. The average age of patients with UC was 45 years, with a median disease duration of 2.5 years. Table 2 presents the clinical baseline characteristics of the study population. Twenty patients (29.2%) achieved endoscopic remission, and 48 patients (70.6%) achieved endoscopic activity. Twenty-three (33.8%) patients with UC exhibited HH, and 45 (66.2%) exhibited HA at baseline (Table 2).

**Table 2.** Clinical baseline characteristics of UC patients.

	*N* = 68	NI ≤ 1 (*n* = 23)	NI > 1 (*n* = 45)	*P*-value
Age (years), mean ± SD	45 ± 14	39 ± 14	48 ± 14	<0.05
Sex, n (%)				<0.01
Female	31 (45.6%)	5 (16.1%)	26 (83.9%)	
Male	37 (54.4%)	18 (48.6%)	19 (51.4%)	
Disease duration (years), median (IQR)	2.5 (4)	2.5 (3)	2.5 (5.5)	0.901
Albumin (g/L), mean ± SD	39.5 ± 3.7	41.0 ± 2.7	38.7 ± 3.9	<0.01
CRP (mg/L), median (IQR)	1.4 (3.4)	1.2 (1.1)	2.6 (5.2)	<0.01
ESR (mm/h), median (IQR)	11 (21)	9 (8)	15 (23)	0.161
NLR, median (IQR)	2.02 (1.45)	2.04 (0.97)	2.61 (1.66)	0.341
EUS-UC score, median (IQR)	4 (3)	3 (2)	5 (3)	<0.001
Truelove and Witt score				<0.01
Remission	15 (22.1%)	11 (73.3%)	4 (26.7%)	
Mild	20 (29.4%)	5 (25.0%)	15 (75.0%)	
Moderate	17 (25.0%)	4 (23.5%)	13 (76.5%)	
Severe	16 (23.5%)	3 (18.8%)	13 (81.3%)	
MES, n (%)				<0.01
Endoscopic remission (≤1)	20 (29.4%)	13 (65.0%)	7 (35.0%)	
Endoscopic Activity (≥2)	48 (70.6%)	10 (20.8%)	38 (69.2%)	
No therapy, n (%) Therapy^a^, n (%)	5 (7.4%)	1 (20.0%)	4 (20.0%)	0.059
Steroids	7 (10.3%)	0	7 (100.0%)	
Biologic therapy^b^	15(22.1%)	8 (53.3%)	7 (46.7%)	

UC, ulcerative colitis; EUS-UC score, Endoscopic Ultrasound- Ulcerative Colitis score; MES, Mayo Endoscopic score; CRP, C-reactive protein; ESR, erythrocyte sedimentation rate; NLR, neutrophil-to-lymphocyte ratio.

^a^All the patients took mesalazine, no patients took immunosuppressants.

^b^Five patients were given infliximab, ten vedolizumab.

### 3.2. The comparison between HH group and activity

**Figure 3.**
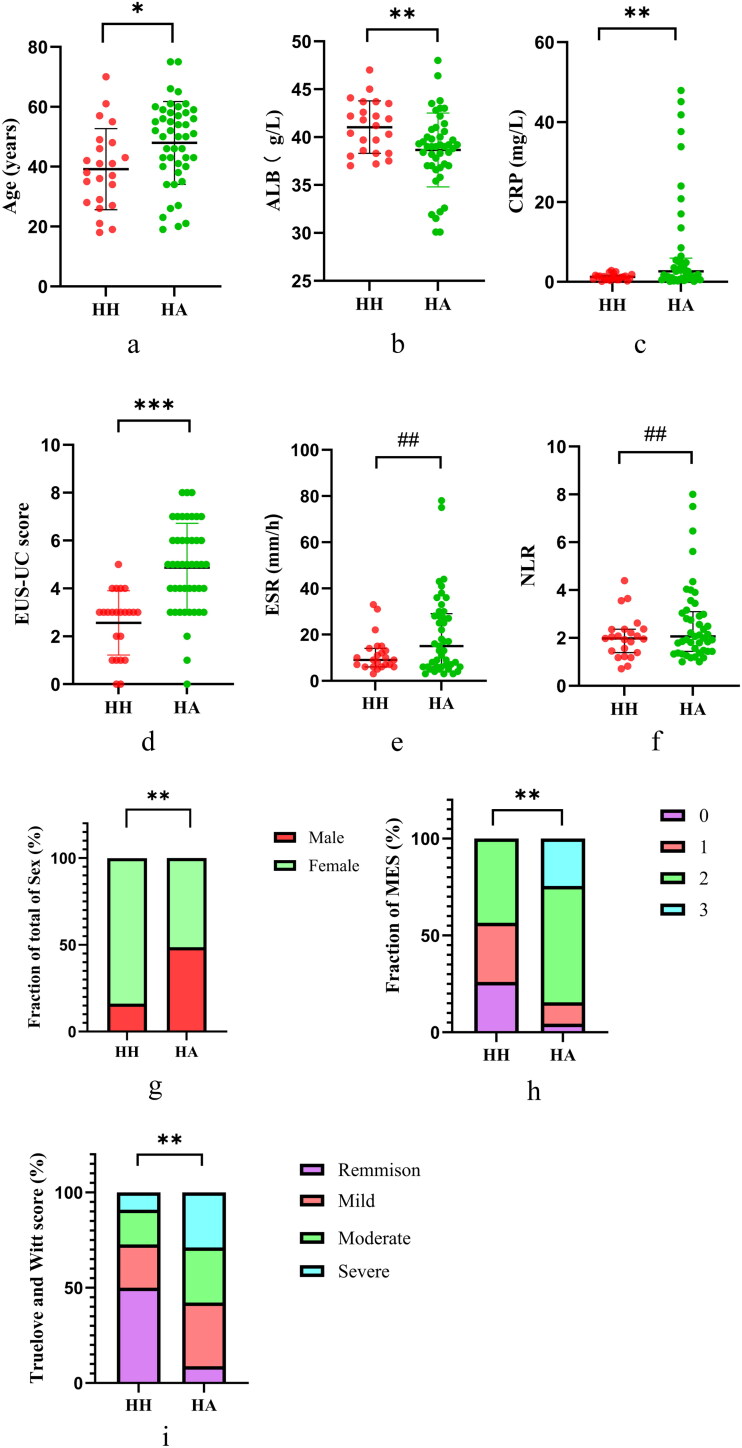
Comparison of various indicators between HH group and HA group: (a) age; (b) ALB; (c) CRP; (d) EUS-UC; (e) ESR; (f) NLR; (g) sex; (h) MES; (i) Truelove and Witt score. *: *p* < 0.05, **: *p* < 0.01, ***: *p* < 0.001, #: *p* > 0.05, ##: *p* > 0.1. EUS-UC score, Endoscopic Ultrasound- Ulcerative Colitis score; MES, Mayo Endoscopic score; CRP, C-reactive protein; ESR, erythrocyte sedimentation rate; NLR, neutrophil-to-lymphocyte ratio; ALB, albumin; HH, histological healing; HA, histological activity.

We defined NI ≤ 1 as HH, and NI ≥ 2 was defined as HA. Based on the aforementioned criteria, we categorized the patients into HH and HA groups. The comparative analysis of these groups is presented in Table 2 and Figure 3. The serum albumin levels in the HH group were higher at 41.0 g/L when compared with the HA group at 38.7 g/L, and both differences were of statistical significance (*p* < 0.05, *p* < 0.01, respectively). In addition, the CRP levels in the HH group were measured at 1.2 mg/L, which is obviously lower than the levels observed in the HA group at 2.6 mg/L (*p* < 0.01). The Truelove and Witt score, MES, and EUS-UC scores in the HH group were significantly lower than those in the HA group, with *p* < 0.01, *p* < 0.01, and *p* < 0.001, respectively. Nevertheless, there existed no statistically significant difference in ESR and NLR levels between the two groups (*p* = 0.161 and *p* = 0.341, respectively).

### 3.3. Relationships between NI score and EUC-UC, MES, truelove and witt score, CRP, albumin, ESR, and NLR

**Figure 4.**
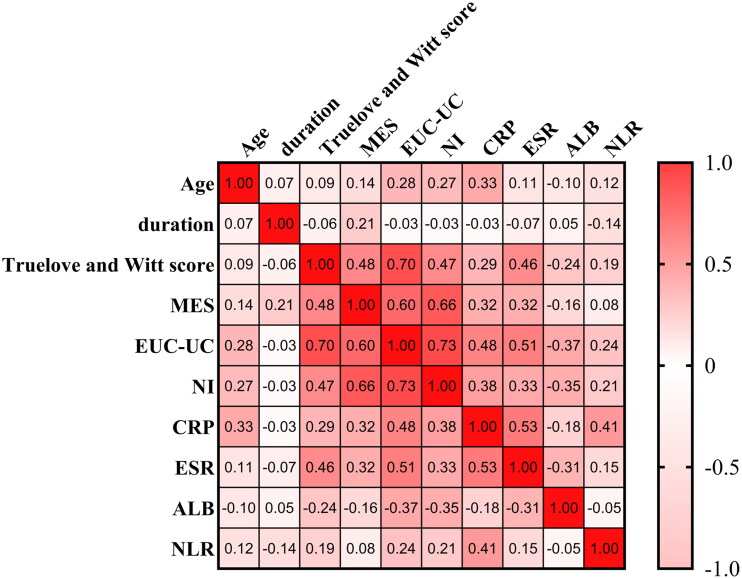
Heatmap of correlation. EUS-UC score, Endoscopic Ultrasound- Ulcerative Colitis score; MES, Mayo Endoscopic score; CRP, C-reactive protein; ESR, erythrocyte sedimentation rate; NLR, neutrophil-to-lymphocyte ratio; NI, Nancy Index; ALB, albumin.

Figure 4 shows the correlation coefficients among various indicators. A strong correlation was observed between the EUS-UC score and the NI (ρ = 0.73, *p* < 0.001), the MES (ρ = 0.51, *p* < 0.001) and the Truelove and Witt score (ρ = 0.70, *p* < 0.001). The EUS-UC score exhibits a moderate association with ESR (ρ = 0.51, *p* < 0.001) and CRP (ρ = 0.48, *p* < 0.001). In addition, our research also found a weak correlation between the EUS-UC score and albumin (ρ=–0.37, *p* < 0.05) and NLR (ρ = 0.24, *p* < 0.05).

We employed NI to evaluate the HH. NI exhibited positive correlations with the MES (ρ = 0.66), the Truelove and Witt scores(ρ = 0.47), ESR(ρ = 0.33), and CRP(ρ = 0.38), while showing a negative correlation with Albumin(ρ=–0.35), all of which were shown to be statistically significant (*p* < 0.001).

### 3.4. Univariable and multivariable logistic regression analysis of variables affecting HH

**Figure 5.**
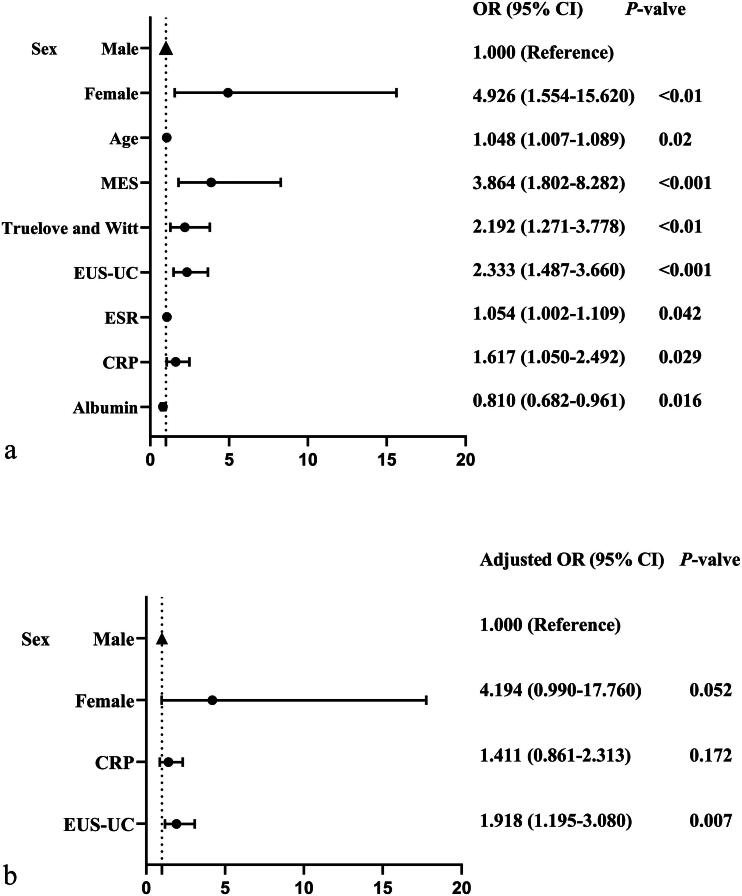
Forest Plot: (a) univariable logistic regression; (b) multivariable logistic regression. EUS-UC, Endoscopic Ultrasound- Ulcerative Colitis score; MES, Mayo Endoscopic score; CRP, C-reactive protein; ESR, erythrocyte sedimentation rate; Truelove and Witt, Truelove and Witt score

Univariable logistic regression revealed several variables associated with HH: age, sex, albumin, CRP, ESR, EUS-UC score, Truelove and Witt score, and MES (*p* < 0.05) (Figure 5a). In the multivariable logistic regression analysis, after adjusting for potential confounders, the EUS-UC score was an independent predictor of HH (Adjusted OR = 1.918, 95% CI: 1.195–3.080, *p* = 0.007). Female sex also showed a trend toward significance (Adjusted OR = 4.194, 95% CI: 0.990–17.760, *p* = 0.052), while CRP was no longer significantly associated with HH (Adjusted OR = 1.411, 95% CI: 0.861–2.313, *p* = 0.172) (Figure 5b).

### 3.5. EUS-UC score, MES, biomarkers and HH in UC patients

**Figure 6.**
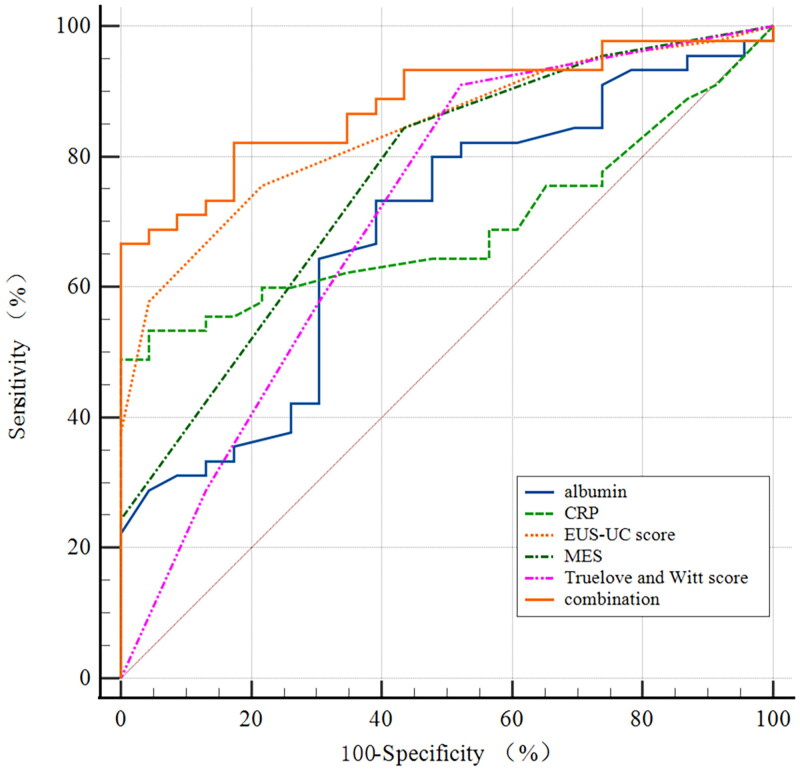
Receiver operating characteristic (ROC) curves of histological healing assessed with MES, EUC-UC score, Truelove and Witt score, CRP, albumin and their combination.

ROC analysis was applied to evaluate the predictive value of the EUC-UC score, the MES and biomarkers in the identification of patients with UC of HA, as shown in Table 3 and Figure 6. The MES showed the AUC of 0.766. Besides, it had a sensitivity of 84.44% and a specificity of 56.52% for the predication of HH (*p* < 0.001). CRP had an AUC of 0.692, a sensitivity of 53.33%, and a specificity of 95.95% (*p* < 0.01) to predict HH. Additionally, EUS-UC demonstrated an AUC of 0.840, a sensitivity of 75.56%, a specificity of 78.26%, and a cutoff value of ≤3 for predicting HH (*p* < 0.01). The Truelove and Witt score exhibits the highest sensitivity rate at 91.11%. By contrast, the CRP demonstrates the highest specificity rate at 95.95%. Furthermore, the Youden index and the AUC for EUC-UC present remarkable values.

**Table 3.** AUC, Sensitivity, specificity and youden index for EUS-UC score, MES, biomarkers and histological activity in patients with UC.

	AUC	CI	Cut-offs	Youden index	Sensitivity	Specificity	*P*-value
MES	0.766	0.662-0.869	>1	0.4097	84.44%	56.52%	<0.001
EUS-UC score	0.840	0.751-0.929	>3	0.5382	75.56%	78.26%	<0.01
CRP	0.692	0.569-0.815	>2.5	0.4899	53.33%	95.95%	<0.01
albumin	0.686	0.553-0.818	≤40	0.3420	73.33%	60.87%	<0.01
NLR	0.571	0.429-0.713	>2.37	0.2261	40.00%	82.61%	0.33
ESR	0.604	0.471-0.738	>15	0.3585	48.89%	86.96%	0.36
Truelove and Witt score	0.713	0.578-0.848	>0	0.3894	91.11%	47.83%	<0.01
combination	0.880	0.801-0.959	───	0.6667	66.67%	100.00%	<0.001

AUC, Area under the receiver operating characteristic curve; MES, Mayo endoscopic score; EUS-UC, Endoscopic Ultrasound-Ulcerative Colitis score, CRP, C-reactive protein; ESR, erythrocyte sedimentation rate; NLR, neutrophil-to-lymphocyte ratio; CI, confidence interval.

### 3.6. The combination of the EUS-UC score and other indices to predict HH

We combined the EUC-UC score, the MES, the Truelove and Witt score, CRP, and albumin to predict HH. The ROC curve which demonstrates the predictive accuracy of this combination is shown in Figure 6. The AUC of the combination was 0.880, with a sensitivity of 66.67% and a specificity of 100.00% for the predication of HH (*p* < 0.001) (Table 3). The combined indicator exhibits a significantly greater sensitivity and specificity relative to EUS-UC, with an AUC surpassing any single indicator.

### 3.7. Post-hoc power analysis

A post-hoc power analysis was conducted based on the observed effect sizes for the primary outcome: the AUC of the EUS-UC score’s ability to predict HH. The study included 68 patients, with 23 having HH and 45 having HA. Using a null hypothesis AUC of 0.500, an observed AUC of 0.840, and an alpha level of 0.05, the analysis exhibited a power of 99% for predicting HH by EUS. In addition, it was indicated that the sample size was sufficient to detect moderate to large effects, ensuring that the results were both statistically significant and generalizable.

### 3.8. Sensitivity analysis and multiple imputation

No significant differences were observed between the included and excluded patients regarding age, sex, disease duration, CRP levels, ESR levels, albumin levels, NLR levels or current medication use (all *p* > 0.05) (Supplement Table 1).

Using multiple imputation methods, our analysis revealed that both the EUS-UC score and MES are significant independent risk factors for predicting HH. The multifactorial regression analysis indicated that EUS-UC has an adjusted OR of 2.123 (95% CI: 1.287-3.502, *p* < 0.01), while MES has an adjusted OR of 2.903 (95% CI: 1.212-6.953, *p* < 0.05) (Supplement Table 2). Moreover, the EUS-UC score demonstrated an area under the AUC of 0.890 (95% CI: 0.837–0.943), with a sensitivity of 80.00% and a specificity of 84.09%, reinforcing its reliability as a diagnostic tool in clinical settings (Supplement Figure 1).

## 4. Discussion

This comprehensive retrospective cohort study revealed positively significant correlations between the EUS-UC score and CRP, ESR, NLR, albumin, the MES and the NI. Furthermore, obviously, the EUS-UC scores in the HH group were significantly lower than those recorded in the HA group. After adjusting for confounders, EUS-UC score was an independent risk factor for HH (adjusted OR = 1.918, 95% CI: 1.195–3.080, *p* = 0.007). When compared with CRP, ESR, NLR, albumin, the Truelove and Witt score and the MES, the EUS-UC score exhibited a superior ability to predict the HH (AUC = 0.840, Sensitivity = 75.56%, Specificity = 78.26%, *p* < 0.01).

Biomarkers have exerted a vital role in the clinical monitoring and treatment decision-making of UC patients [22], serving as a noninvasive, convenient, and cost-effective approach, and are acknowledged as midterm treatment targets [2]. CRP is a recognized biomarker for evaluating inflammatory processes [23], and a meta-analysis performed by Mosli et al. indicated that it could predict endoscopic inflammation in patients with UC [24]. Our study showed a correlation between CRP and endoscopic activity, which is similar to previous research findings. Although previous studies lacked evidence linking CRP to HH, our investigation found a modest association with NI; CRP levels below 2.5 mg/L could predict HH, with an AUC of 0.692, sensitivity of 53.33%, and specificity of 95.95%, indicating CRP’s potential as a biomarker for the evaluation of HH.

As indicated by STRIDE II, EH has been a long-term outcome in UC patients [2]. Numerous studies have indicated that achieving EH can enhance long-term outcomes by decreasing the need for corticosteroids, and reducing the occurrence of recurrences, hospitalizations, colectomies, and colorectal tumors [25]. The MES and the Ulcerative Colitis Endoscopic Index of Severity (UCEIS) are frequently adopted for the evaluation of EH, where EH is defined as MES = 0 or UCEIS ≤ 1. Our study suggested that the EUC-UC score was significantly associated with the MES, finding that the EUC-UC score can be used to assess endoscopic activity effectively. Nevertheless, studies which document the correlation between EUS and endoscopic findings are scarce. Soweid et al. identified a strong relationship between bowel wall thickening and mucosal appearance by using catheter-probe-assisted endoluminal ultrasonography [26]. In a separate study, Rana and colleagues discovered a significant correlation between mucosal and submucosal thickness and endoscopic severity scores in patients with UC [27]. In recent years, a Chinese study revealed a significant positive correlation between the EUS-UC score and the Truelove and Witt scores, the Mayo score, and the UCEIS, indicating the potential for monitoring treatment response [18]. In summary, the findings of the present study are in consistence with prior research, suggesting a positive correlation between EUS and endoscopic outcomes.

In UC, HH, a more advanced form of mucosal healing, requires the disappearance of active inflammatory cells in mucosal biopsies and has emerged as a therapeutic goal for preventing long-term complications [2,28]. Endoscopy and biopsy remain the gold standards for assessing histological pathology. However, mucosal biopsies require invasive endoscopic investigations [29], and local biopsies are inadequate for detecting deeper lesions of the intestinal wall and the entire colon [4]. Therefore, an increasing number of examinations are being validated as substitutes for biopsies. Fecal calprotectin, intestinal ultrasound, transperineal ultrasound and narrow-band imaging endoscopy have reported to be significantly related to HH [2,30–32]. Moreover, previous studies have demonstrated that EUS can detect inflammation in UC patients [15,16]; particularly, increased rectal wall thickness is directly proportional to histological severity [33]. Since the EUS-UC score was proposed, only a few studies have focused on exploring its relevance to clinical and endoscopic activity [18]. Moreover, no articles have elucidated its association with HH. However, our study is the first to demonstrate the relationship between the EUS-UC score and HH. In this study, the total EUS-UC score for the HA group was significantly higher than that of the HH group, and Spearman’s correlation analysis suggested that the EUS-UC score showed a strong relationship to HH. Owing to the strong relationship between the EUS-UC score and histological outcomes, reducing the number of biopsies to some extent can contribute to decreasing complications including perforation and bleeding.

Our study found several factors linked to HH in UC patients, with the EUS-UC score identified as a strong independent predictor. To ensure that our findings are reliable, we conducted multivariable logistic regression analyses while adjusting for potential confounders, the EUS-UC score continued to reveal a significant association with HH, underscoring its effectiveness as a reliable marker for HH. However, we must recognize that unmeasured confounders may impact our results. Moreover, further research should include a wider variety of potential confounders to better understand the predictors of HH in UC.

This research also revealed that the EUS-UC score is a superior predictor of HH compared with other indices, with an AUC 0.840, specificity 78.26%, sensitivity 75.56%, and EUS-UC score ≤ 3 indicating HH. These results are more effective than the current biomarkers and endoscopic indices for the measurement of HH in UC. The superior performance of EUS-UC in predicting HH may be attributed to several factors. At first, EUS enables direct visualization and measurement of intestinal wall layers. This provides insights into submucosal and muscularis propria changes, which of particular importance because HH includes not only the recovery of the mucosal layer but also the restoration of the deeper layers [2,34]. Second, EUS can detect subtle vascular changes through Doppler imaging that may reflect underlying microvascular normalization, which is a key component of true HH [35]. This capability surpasses conventional endoscopy, primarily assessing superficial mucosal features. Thirdly, the quantitative nature of EUS measurements (e.g. wall thickness, layer pattern) provides objective, reproducible data that may be less susceptible to inter-observer variability than subjective endoscopic scoring systems. EUS-UC score exactly integrated intestinal wall thickness, the depth of inflammation, and hyperemia, making it a strong predictor for HH.

However, the limited ability of CRP and albumin to predict HH, in relative to the EUS-UC score, arises because CRP is an acute-phase reactive protein produced by the liver that typically rises during acute inflammation or infection [36], while albumin suggests intestinal inflammation and reflects nutritional status [37]. This indicates that both markers are not specific to inflammation when predicting UC and can be affected by various other factors. Inflammation in UC mostly occurs in the mucosal layer and submucosal layer. However, endoscopy can only observe the surface of the mucosa, and it is challenging to accurately evaluate the condition below the mucosa, resulting in a low predictive HH capability of the MES [38].

While bowel ultrasound is a non-invasive tool for the assessment of disease activity in UC, which is increasingly recognized as a valuable method, endoscopic ultrasound provides superior spatial resolution and precise localization of pathological changes. EUS is particularly valuable in assessing the depth and extent of inflammation, differentiating between inflammatory and fibrotic changes, and detecting early complications [17]. EUS shows superior performance in minimizing interference from intestinal peristalsis and intraluminal gas, which are common limitations of transabdominal ultrasound [39]. The direct contact between the EUS probe and the intestinal wall significantly decreases artifacts caused by these factors, allowing for more reliable and consistent imaging [40]. The choice between these modalities should be based on the specific clinical question, with EUS being particularly useful in complex cases which require detailed structural assessment. This study did not include abdominal ultrasound examinations, which precluded direct comparison between these two modalities. Moreover, future studies should consider a head-to-head comparison of EUS and abdominal ultrasound to better establish their respective roles in UC evaluation.

The combining targets should be used as a strategy to improve outcomes in UC patients in accordance with the STRIDE II [2]. Therefore, we integrated the EUC-UC score and MES, CRP, albumin and the Truelove and Witt score to predict HH. The AUC of the combination was 0.880, with a sensitivity of 66.67% and a specificity of 100.00%. Our analysis revealed significant improvements in the sensitivity and specificity of the combined indicators. This combination can accurately predict HH outcomes and enhance the reliability of diagnostic assessments in clinical practice.

The robustness of our findings was validated through sensitivity analyses and multiple imputation methods, addressing potential biases from missing data and strengthened the reliability of our conclusions. Our sensitivity analysis revealed no significant differences in baseline characteristics between included and excluded patients (all *p* > 0.05). This suggested that the exclusion of incomplete data did not introduce substantial bias. However, it is vital to acknowledge that unmeasured confounders may still exist and could potentially influence the results. After multiple imputation, EUS-UC score was still confirmed as an independent risk factor in the multivariable logistic regression analysis, with the adjusted OR rising from 1.918 to 2.123, therefore demonstrating its robustness. In the meanwhile, the multiple imputation analysis caused an increase in the AUC value from 0.840 to 0.890, indicating improved diagnostic performance of the EUS-UC score in predicting HH. Moreover, this improvement suggests that the original analysis may have underestimated the model’s performance caused by missing data. By accounting for the uncertainty introduced by missing data, the multiple imputation approach provided a more robust estimate of the EUS-UC score’s diagnostic accuracy. In addition, the higher AUC value (0.890) further supports the clinical utility of the EUS-UC score as a reliable tool for predicting HH in UC patients.

However, our study still had the following limitations. First, the small sample size and single-center design may limit generalizability. Besides, future studies should expand to multiple centers with larger cohorts. Secondly, we used only the NI for histological assessment, as other indices including the Robart Histological Index (RHI) or Geboes score lack thorough validation in UC [41,42]. Accuracy would be improved by incorporating multiple histological indices [21]. Thirdly, due to financial and logistical constraints, we could not include biomarkers like fecal calprotectin and leucine-rich alpha-2 glycoprotein, which should be integrated in future studies for a more comprehensive evaluation. Fourthly, EUS-UC scores were assigned by a single specialist, ensuring consistency but missing inter-observer variability. Future studies should involve multiple raters in order to assess reliability. Fifthly, the retrospective and cross-sectional design prevented assessment of long-term HH persistence, relapse rates, or late responders. Longitudinal studies with defined follow-up intervals are essential to evaluate EUS-UC’s utility in the prediction of sustained remission and relapse risk. Sixthly, the absence of a control group (e.g. healthy individuals or non-UC patients) limits the ability to establish baseline EUS scores and validate specificity for UC. In further studies, healthy controls and non-UC patients should be involved to establish baseline EUS scores for better validation of the EUS-UC score’s specificity. Seventhly, the lack of analysis on data variability and outliers may influence subgroup applicability, though logistic regression confirmed EUS-UC score as an independent predictor of HH. Finally, the retrospective design may introduce selection bias, as sicker patients were more probably to undergo EUS and histological assessment. Future prospective studies with standardized protocols are needed to deal with these biases and validate our findings. In addition, sector scanning EUS is technically challenging and should be performed by experienced physicians.

## 5. Conclusions

The EUS-UC score shows a significant positive association with both the endoscopic activity and the HA. Moreover, the EUS-UC score demonstrates its potential as an effective scoring system for predicting HH. Future research should concentrate on validating the EUS-UC score as a reliable tool for predicting clinical recurrence and evaluating remission rates across different drug treatments.

## Supplementary Material

Supplement Material.docx

supplement figure.tif

## Data Availability

The data supporting the findings of this study can be requested from the corresponding author.
