# Supplementary material for: Role of endoscopic ultrasound as a predictor of histological healing in ulcerative colitis
Source: Ann Med. 2025 Apr 30;57(1):2499961. doi: 10.1080/07853890.2025.2499961 (PMC12044909; doi:10.1080/07853890.2025.2499961)
Supplement: Supplement Material.docx [file IANN_A_2499961_SM4836.docx]

**Supplement Tables with Captions**

**Supplement Table 1** Comparison between inclusion and exclusion groups.

| Clinical characteristic | Inclusion (n=68) | Exclusion (n=56) | *P*-value |
| --- | --- | --- | --- |
| Sex |  |  | 0.77 |
| Male | 31 (45.6%) | 27 (48.2%) |  |
| Female | 37 (54.4%) | 29 (51.8%) |  |
| Disease duration (years)，median(range) | 2.5 (0.5-30) | 3 (0.5-20) | 0.11 |
| Age (years), mean ±SD | 45±14 | 44±16 | 0.37 |
| Albumin (g/L), mean ±SD | 39.5±3.7 | 38.2±5.6 | 0.16 |
| CRP (mg/L), median (IQR) | 1.4 (3.4) | 2.9 (7.0) | 0.08 |
| ESR (mm/h), median (IQR) | 11 (21) | 17 (24) | 0.08 |
| NLR, median (IQR) | 2.02 (1.44) | 2.48 (2.23) | 0.12 |
| EUS-UC score, median (IQR) | 4 (3) | 4 (3) | 0.93 |
| Disease extent |  |  | 0.17 |
| Extensive | 11.8% | 30.4% |  |
| Left-sided | 48.5% | 50.0% |  |
| Proctitis | 39.7% | 19.6% |  |
| Truelove and Witt score |  |  | 0.34 |
| Remission | 22.1% | 22.2% |  |
| Mild | 29.4% | 18.5% |  |
| Moderate | 25.0% | 38.9% |  |
| Severe | 23.5% | 20.4% |  |
| Mayo endoscopic score |  |  | 0.94 |
| Endoscopic remission (≤1) | 28.0% | 28.6% |  |
| Endoscopic Activity (≥2) | 72.0% | 71.4% |  |
| Nancy index |  |  | 0.67 |
| Histological healing (≤1) | 33.8% | 37.5% |  |
| Histological activity (≥2) | 66.2% | 62.5% |  |
| No therapy  Therapy ^a^ | 7.4% | 8.9% | 0.75 |
| Steroids | 10.3% | 10.3% | 0.70 |
| Biologic therapy ^b^ | 22.1% | 26.8% | 0.82 |

EUS-UC score, Endoscopic Ultrasound- Ulcerative Colitis score; MES, Mayo Endoscopic score; CRP, C-reactive protein; ESR, erythrocyte sedimentation rate; NLR, neutrophil-to-lymphocyte ratio; ALB, albumin.

^a^ All the patients took mesalazine, no patients took immunosuppressants

^b^ Five patients were given infliximab, ten vedolizumab

**Supplement Table 2** Univariable and multivariable logistic regression analysis for each variable of imputed data.

| Variables | Univariable Logistic Regression | | Multivariable Logistic Regression | |
| --- | --- | --- | --- | --- |
|  | OR (95% CI) | *P*-value | Adjusted OR (95% CI) | *P*-value |
| Age | 1.030 (1.000-1.061) | 0.049 |  |  |
| Sex |  |  |  |  |
| Male | 1.000 (Reference) |  | 1.000 (Reference) |  |
| Female | 4.273 (1.892-9.650) | <0.001 | 3.167 (0.966-10.385) | 0.057 |
| Albumin | 0.863 (0.777-0.960) | <0.01 |  |  |
| CRP | 1.100 (1.018-1.193) | <0.05 | 1.105 (0.985-1.239) | 0.089 |
| ESR | 1.055 (1.002-1.109) | <0.01 |  |  |
| EUS-UC | 3.267 (2.083-5.126) | <0.001 | 2.123 (1.287-3.502) | <0.01 |
| TW | 2.192 (1.271-3.778) | <0.01 |  |  |
| MES | 7.502 (3.612-15.580) | <0.001 | 2.903 (1.212-6.953) | <0.05 |
| NLR | 4.582 (2.680-7.835) | <0.001 |  |  |

OR: Odds Ratio, CI: Confidence Interval; EUS-UC score, Endoscopic Ultrasound- Ulcerative Colitis score; MES, Mayo Endoscopic score; CRP, C-reactive protein; ESR, erythrocyte sedimentation rate; NLR, neutrophil-to-lymphocyte ratio; ALB, albumin; TW, Truelove and Witt score

**Supplement Figure Caption**

**Supplement Figure 1** ROC of EUS score after multiple imputation

EUS-UC score, Endoscopic Ultrasound- Ulcerative Colitis score
