# Supplementary figures and images for: Role of endoscopic ultrasound as a predictor of histological healing in ulcerative colitis
Source: Ann Med. 2025 Apr 30;57(1):2499961. doi: 10.1080/07853890.2025.2499961 (PMC12044909; doi:10.1080/07853890.2025.2499961)

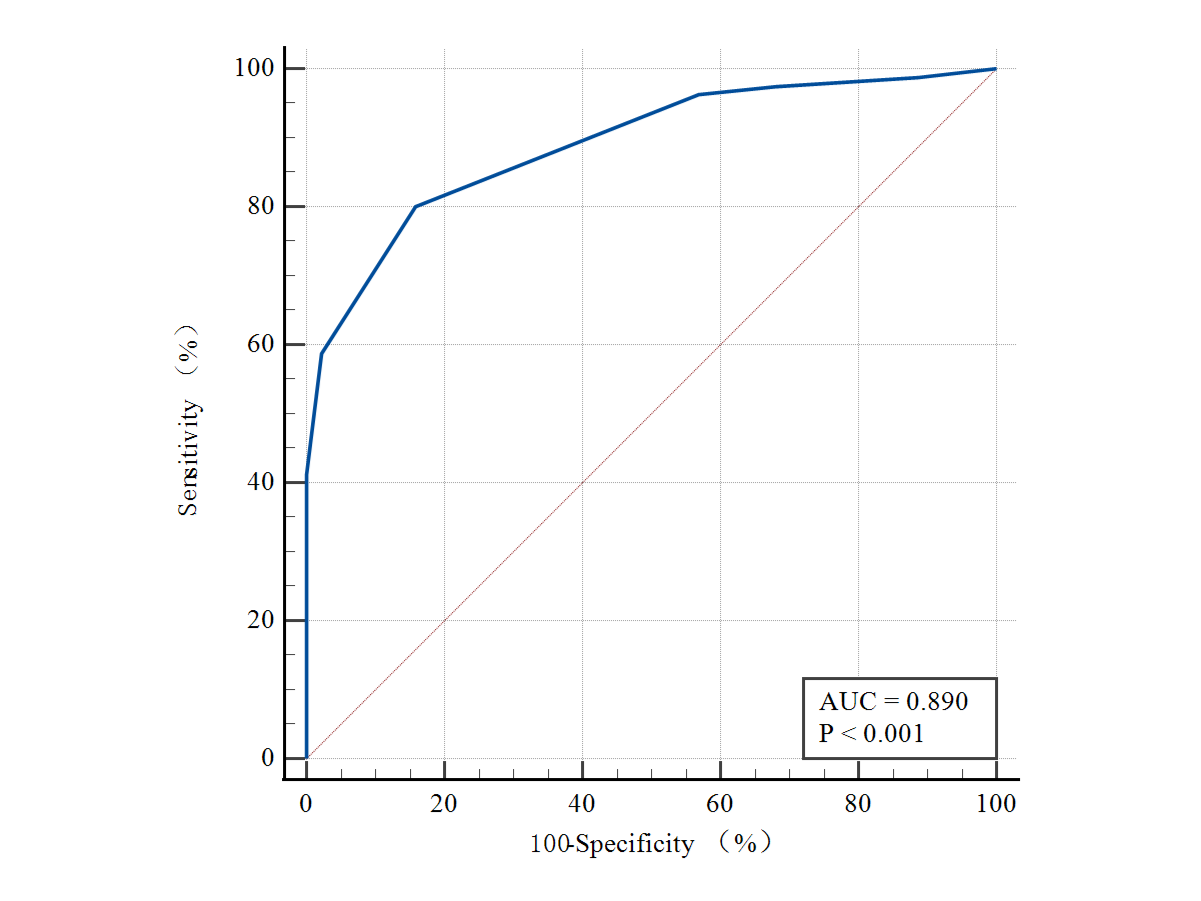

Supplement: supplement figure.tif [file IANN_A_2499961_SM4835.tif]
